# Feeder-free ex vivo expansion of cord blood-derived natural killer cells for enhanced proliferation and functional maturation

**DOI:** 10.1038/s41598-026-41101-5

**Published:** 2026-02-24

**Authors:** Isabel Doutor, Gabriel Costa, Beatriz Filipe, Carla Lilaia, Pedro Fonte, Ana Fernandes-Platzgummer

**Affiliations:** 1 iBB-Institute for Bioengineering and Biosciences, Department of Bioengineering and Associate Laboratory i4HB-Institute for Health and Bioeconomy, Instituto Superior Técnico, Universidade de Lisboa, 1049-001 Lisbon, Portugal; 2 Hospital São Francisco Xavier, Centro Hospitalar de Lisboa Ocidental, 1449-005 Lisbon, Portugal; 3 Centro de Ciências do Mar do Algarve (CCMAR/CIMAR LA), Universidade do Algarve, Gambelas Campus, 8005-139 Faro, Portugal; 4 Department of Chemistry and Pharmacy, Faculty of Sciences and Technology, Universidade do Algarve, Gambelas Campus, 8005-139 Faro, Portugal; 5 Present Address: NTT Data Portugal, 1050-094 Lisbon, Portugal

**Keywords:** Cord blood (CB), Natural killer (NK) cells, Expansion, Cytotoxicity, Cell manufacturing, Immunotherapy, Biotechnology, Cancer, Immunology, Oncology

## Abstract

Umbilical cord blood (CB)-derived natural killer (NK(CB)) cells are a promising immunotherapeutic modality due to their cytotoxicity, allogeneic compatibility, and reduced immunogenicity relative to peripheral blood NK cells (NK(PB)). Although several commercial culture media have been optimized for NK(PB) expansion, their suitability for NK(CB) cells remains insufficiently characterized. This study compares the expansion efficiency, phenotypic shifts, functional activation, and cost-effectiveness of NK(CB) cells expanded ex vivo under feeder-free static culture conditions with IL-2-supplemented culture media. CB-derived CD56⁺ cells isolated by magnetic-activated cell sorting were cultured in six candidate culture media, after which CTS™ NK-Xpander™ (NKX) and NK MACS^®^ (NKM) with 1% (NKM1) or 2% (NKM2) supplement were selected for further evaluation. NKX achieved the highest average fold expansion (18.8 ± 7.0), followed by NKM2 (16.8 ± 7.3) and NKM1 (14.3 ± 3.5). All conditions increased cytotoxicity and CD107a degranulation, with NKX consistently yielding stronger functional responses. Phenotypic analysis revealed a shift toward a CD56^bright^CD16⁻ profile, particularly in NKM cultures, accompanied by cytokine-associated CD16 downregulation. Cost analysis identified NKX and NKM1 as the most cost-efficient conditions, whereas NKM2 doubled manufacturing costs due to higher supplement requirements. In conclusion, NKX and NKM media effectively support feeder-free ex vivo expansion of NK(CB) cells with high viability and robust functional activation. Phenotypic changes, donor-dependent variability, and CD16 downregulation should be considered when designing clinically translatable manufacturing strategies. These findings are essential to enhance scalability, reduce manufacturing costs, and advance NK(CB) cells as an accessible and effective immunotherapy, facilitating their integration into clinical-grade manufacturing and scalable bioprocessing pipelines.

**Supplementary Information:**

The online version contains supplementary material available at 10.1038/s41598-026-41101-5.

## Introduction

Natural killer (NK) cells are innate lymphocytes that play a critical role in immune surveillance by eliminating virally infected and malignant cells through cytolytic granules. Unlike T cells, NK cells can lyse target cells without prior activation or human leukocyte antigen restriction, making them highly attractive candidates for cancer treatment^1^. Umbilical cord blood (CB) is an attractive source of NK cells for clinical applications due to its accessibility and ethical acceptability as a non-invasive medical waste product^2,3^. Additionally, CB is a source already established for hematopoietic stem cell transplantation, further supporting its suitability for developing off-the-shelf cell-based therapies^3,4^. CB-derived NK cells (NK(CB)) exhibit superior proliferative potential and lower immunogenicity compared to peripheral blood (PB)-derived NK cells (NK(PB))^5^, making them well-suited for allogeneic therapies. Clinical infusions of NK(CB) cells are well tolerated, carry minimal graft-versus-host disease (GvHD) risk, and have induced complete remission in poor-prognosis acute myeloid leukemia patients^6,7^. This enhanced tolerance is linked to their distinct receptor repertoire, characterized by reduced diversity of inhibitory KIRs and a higher proportion of NKG2A⁺ cells, which collectively lower alloreactive potential. Additionally, NK(CB) cells exhibit lower basal levels of key activating receptors and retain strong cytokine responsiveness, contributing to their lower intrinsic alloreactivity compared with NK(PB)^3^. Multiple trials have explored their use across hematologic and solid malignancies, and their ability to be cryopreserved supports the development of off-the-shelf immunotherapy products^8^.

Efficient in vitro expansion of NK cells is critical for clinical translation^9^. Expansion protocols vary widely in NK cell source^10,11^, cytokine supplementation^12,13^, and the use of feeder cells or artificial antigen-presenting cells^14,15^. Regardless of the source, expanded NK cells typically exhibit increased cytotoxicity^16^, even against NK-resistant tumors^13^. Culture media composition is another key factor that influences NK cell proliferation and function. Although several commercial formulations are available, most were originally optimized and benchmarked for NK(PB) cells^17,18^, leaving a significant knowledge gap regarding their performance with NK(CB) cells. Optimizing NK(CB) culture conditions is also crucial for large-scale production under good manufacturing practice (GMP) settings. Many current expansion strategies remain difficult to scale and standardize, limiting their clinical applicability. Recent advances in NK cell-based immunotherapy, particularly the emergence of CB-derived chimeric antigen receptor (CAR)-engineered NK cells (CAR-NK(CB)) underscore the need for efficient and cost-effective expansion methods^19^. Compared to CAR-T cells, CAR-NK(CB) cell-based products offer important advantages, including lower risk of severe cytokine release syndrome^20^, the possibility of cryopreservation as universal off-the-shelf therapies, and the potential for large-scale manufacturing from a single CB donation^2,3^. These unique features have fueled growing interest in CAR-NK cell-based therapies for hematologic malignancies, solid tumors, and even autoimmune diseases^21^.

This study addresses this critical gap by evaluating the expansion of NK(CB) cells in commercially available culture media, focusing on their impact on NK(CB) cell proliferation, phenotype, and functional activation. By refining NK(CB) expansion strategies, we aim to establish a robust foundation for scalable, clinically compliant manufacturing, accelerating the development of next-generation NK cells-based immunotherapies for both hematological and solid cancers.

## Materials and methods

### Mononuclear cell isolation

Umbilical cord blood units were obtained from healthy donors at Hospital São Francisco Xavier, Centro Hospitalar de Lisboa Ocidental (Lisbon, Portugal), upon informed consent. The study was approved by the Ethics Committee of Centro Hospitalar de Lisboa Ocidental (CHLO), and all methods were performed in accordance with the relevant guidelines and regulations. CB samples (70–140 mL) were diluted with phosphate-buffered saline (PBS; Thermo Fisher Scientific) with 2 mM ethylenediaminetetraacetic acid (EDTA; Sigma-Aldrich) (PBS-EDTA) and layered onto Ficoll-Paque™ (Cytiva) for density gradient centrifugation (400 × g, 30 min, swing bucket rotor, brake off). The buffy coat was collected, washed with PBS-EDTA and treated with 155 mM ammonium chloride (4 °C, 10 min). Mononuclear cells from CB (MNC(CB)) were counted, cryopreserved in low-glucose Dulbecco’s Modified Eagle Medium (DMEM; Thermo Fisher Scientific) supplemented with 10% (v/v) fetal bovine serum (FBS; Thermo Fisher Scientific), 1% (v/v) antibiotic-antimycotic (A/A; 10,000 units/mL of penicillin, 10,000 µg/mL of streptomycin and 25 µg/mL of Amphotericin B, Thermo Fisher Scientific) and 10% (v/v) dimethyl sulfoxide (DMSO; Sigma-Aldrich), and stored in a liquid nitrogen tank.

### CD56^+^ cell enrichment

MNC(CB) were thawed in DMEM supplemented with 10% (v/v) FBS and 1% (v/v) A/A. Prior to CD56^+^ cell isolation by magnetic-activated cell sorting (MACS), a pre-enrichment characterization was performed to determine the percentage of CD3^−^CD56^+^ cells by flow cytometry (CD3 PerCP-Cy5.5 (BioLegend) and CD56 PE (BioLegend)). CD56^+^ cells were isolated using MACS with CD56 MicroBeads (Miltenyi Biotec), following the manufacturer instructions, and MACS efficiency was calculated using Eq. 1. Briefly, MNC(CB) were resuspended in PBS supplemented with 0.5% (v/v) bovine serum albumin (Sigma-Aldrich) and 2 mM EDTA (MACS Buffer). The cell suspension was then incubated with magnetic anti-CD56 microbeads. Afterwards, MNC(CB) were washed with MACS Buffer and loaded into an LS column (Miltenyi Biotec). After washing and collecting the negative fraction, the column was removed from the magnet and the positive fraction was flushed and recovered. The CD56-enriched fraction was further characterized in terms of cell fitness, immunophenotypic/activation profile, cytotoxic, degranulation and proliferative capacity.

1 $$\:\%\:CD56\:MACS\:Efficiency=\:\frac{{(Cell\:Number\:\times\:\:{Percentage\:of\:CD56}^{+}\:Cells)}_{After\:{CD56}^{+}\:Enrichment}}{{(Cell\:Number\:\times\:\:{Percentage\:of\:CD56}^{+}\:Cells)}_{Before\:{CD56}^{+}\:Enrichment}}\times\:100$$

### Ex vivo expansion of CD56^+^ cells

CD56^+^-sorted cells were cultured at 1 × 10^6^ cells/mL in 24-well plates using different commercial culture media: CTS™ NK-Xpander™ (Thermo Fisher Scientific), GMP SCGM (Sartorius CellGenix GmbH), NK MACS^®^ (Miltenyi Biotec), PRIME-XV NK Cell CDM (FUJIFILM Irvine Scientific), StemSpan™ SFEM II (STEMCELL Technologies), and X-VIVO™ 15 (Lonza) (Table S1). CTS™ NK-Xpander™ and NK MACS^®^ were supplemented with additives recommended by the manufacturers. An additional condition was evaluated for NK MACS^®^, where it was supplemented with 2% (v/v) of the corresponding supplement^17,22^. When specified, culture media were also supplemented with 5% (v/v) human serum (hS; Sigma-Aldrich). Interleukin-2 (IL-2; PeproTech) was added at 500 U/mL for CTS™ NK-Xpander™ and NK MACS^®^, following the manufacturer recommendations. For the serum-free culture media GMP SCGM, PRIME-XV NK Cell CDM, StemSpan™ SFEM II, and X-VIVO™ 15, a higher concentration of 1000 U/mL was used, in line with IL-2 concentrations commonly reported for primary NK cell expansion in the literature, and consistent with the manufacturers specifications for serum-free use^23–25^. Cell expansion was conducted for approximately 27 days at 37 °C and 5% CO_2_ in a humidified atmosphere. The culture medium was refreshed every 2–3 days by adding fresh complete medium and adjusting the cell density to 1 × 10^6^ cells/mL. Total cumulative costs during expansion were calculated by considering the complete culture media used, and the corresponding fold increases obtained with each medium.

### Analysis of metabolites concentration

To monitor glucose and lactate levels, the supernatant from NK cell cultures was collected each time the medium was replaced and centrifuged at 360 × g for 10 min. The concentrations of glucose and lactate were measured using the YSI 2500 Biochemistry Analyser (Yellow Springs Instrument), which employs membrane-bound immobilized enzyme quantification.

### Growth kinetics analysis

The maximum specific growth rate (µ_max_, day^−1^) was determined during the exponential growth phase, assuming negligible cell death. µ_max_ was calculated according to Eq. (2), where X_v_ represents the viable cell concentration at time t. Values of µ_max_ were obtained from the slope of the linear regression of log-transformed X_v_ over the experimentally defined exponential growth interval.

2 $$\:\frac{d{X}_{v}}{dt}={\mu\:}_{max}\times\:{X}_{v}$$

The doubling time (t_d_, days), was derived from µ_max_ using Eq. (3):

3 $$\:{t}_{d}=\frac{\mathrm{l}\mathrm{n}\left(2\right)}{{\mu\:}_{max}}$$

### Apoptosis detection

Viable, apoptotic, and necrotic cell populations were quantified after each cell sorting using the FITC-Annexin V Apoptosis Detection Kit (BD Biosciences), according to manufacturer instructions. Briefly, cells were washed with PBS, resuspended in Annexin V Binding Buffer, and incubated with FITC-Annexin V and propidium iodide (PI). After staining, cells were analyzed by flow cytometry using a BD FACSCanto™ (BD Biosciences). Data were processed and analyzed using FlowJo v10 software (FlowJo LLC).

### Immunophenotype

The immunophenotype of NK(CB) cells was assessed immediately after cell sorting and at various time points during expansion. The gating strategy is shown in Fig. S1. Briefly, isolated cells were washed with PBS and incubated with LIVE/DEAD™ Fixable Far Red Dead Cell Stain Kit (L/D; Invitrogen) at room temperature (RT) for 15 min. After washing, cells were stained with the following pre-titrated mouse anti-human monoclonal antibodies: CD3 PerCP-Cy5.5, CD56 PE, and CD16 FITC (BioLegend) for 15 min at RT. For activation/inhibitory profiling, KIR2DL2/L3 FITC (BioLegend) and NKp44 APC (BioLegend) were used. Cells were analyzed by flow cytometry using a BD FACSCalibur™ or BD FACSCanto™ (BD Biosciences). Samples from each experimental batch were acquired on the same instrument. No quantitative comparisons of fluorescence intensity across instruments were performed. All percentage-based analyses were conducted within samples acquired on the same cytometer, and no biological conclusions were drawn from comparisons across instruments. Data were analyzed using FlowJo v10 (FlowJo LLC).

### Cytotoxicity assay

The K562 cell line was cultured in Advanced Roswell Park Memorial Institute (RPMI) 1640 medium (Thermo Fisher Scientific) supplemented with 10% FBS, 2 mM GlutaMAX (Thermo Fisher Scientific), and 1% A/A, at 37 °C with 5% CO_2_. The culture medium was renewed every 2–3 days, maintaining a cell density of approximately 1 × 10^6^ cells/mL. K562 cells (target cells) were labeled with bis(acetoxymethyl) 1,4,7,10-tetraazacyclododecane (BATDA; Perkin Elmer) and co-cultured with NK cells (effector cells), immediately after sorting and on day 20 of expansion, in 96-well U-bottom plates, in triplicates, for 3 h at different effector-to-target (E:T) ratios (1:1, 5:1, 10:1, 20:1, and 50:1). After co-incubation, the supernatant from each well was collected and mixed with europium solution (Perkin Elmer) under shaking in the dark for 15 min. Fluorescence was measured using a Tecan Infinite 200 PRO multilabel plate reader (Tecan Austria GmbH). Cytotoxicity was calculated using Eq. (4).

4 $$\:\%\:Specific\:Lysis=\:\frac{Lysis-Spontaneous\:Lysis}{Maximum\:Lysis-Spontaneous\:Lysis}\times\:100$$

### Degranulation assay

CD56^+^-enriched cells and day 20-expanded cells were co-cultured with K562 (target cells) in 96-well U-bottom plates at the E:T ratio of 10:1 for 3 h^26^. Cells were then stained with anti-human CD107a FITC (BD Biosciences) and incubated for an additional 2 h in the presence of Monensin/GolgiStop™ (BD Biosciences). After incubation, cells were stained with L/D, CD3 PerCP-Cy5.5, and CD56 PE, followed by flow cytometry analysis.

### Statistical analysis

Results are presented as the mean ± standard error of the mean (SEM). Data normality was assessed using the Shapiro–Wilk test. The number of independent experiments, statistical tests, and statistical significance levels are described in figures captions. All analysis were performed using GraphPad Prism 9 software (Dotmatics).

## Results

### Efficient recovery of viable CD56⁺ NK cells from cryopreserved MNC(CB) using MACS

**Fig. 1:**
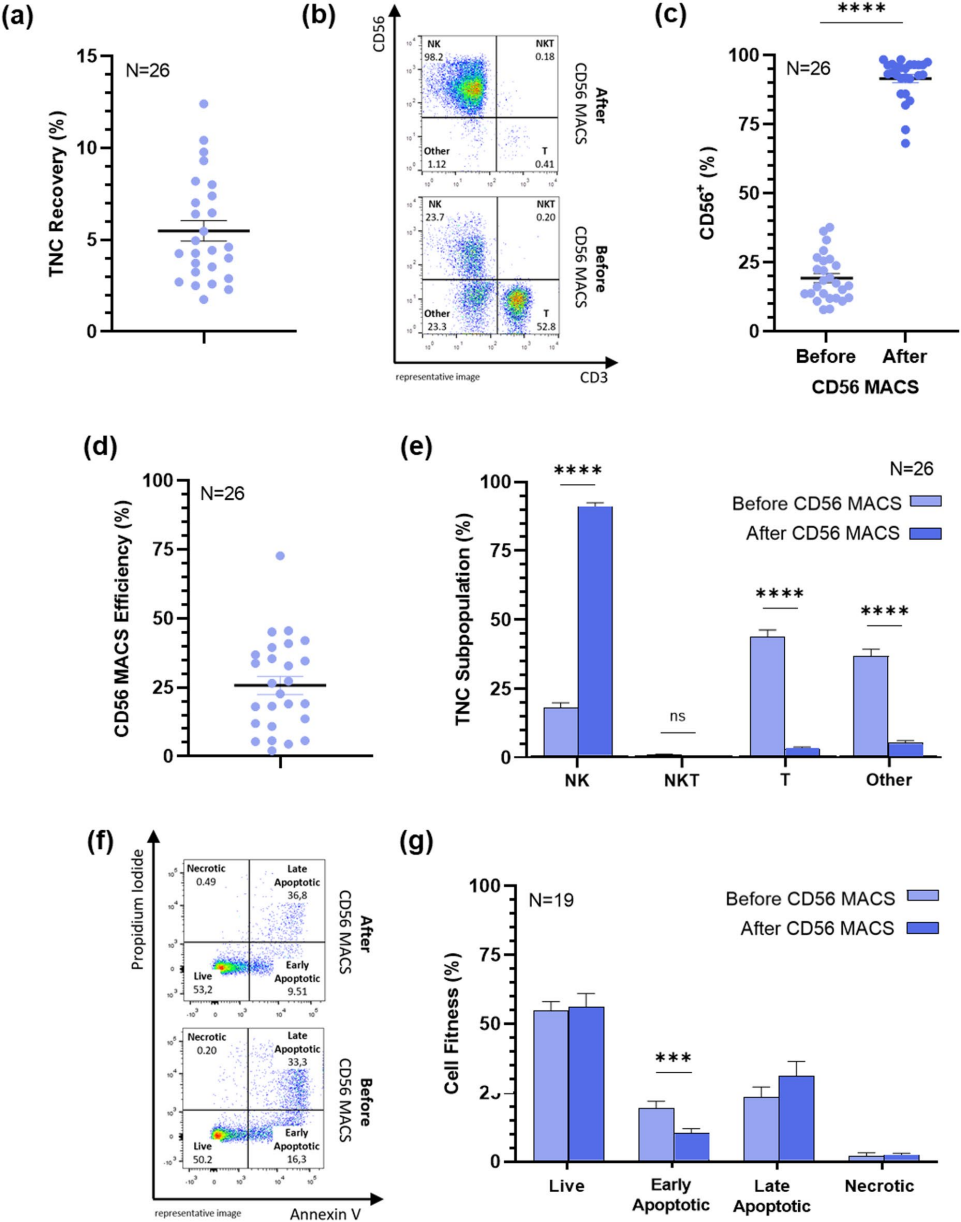
CD56^+^ cell enrichment and apoptosis assay. MNC(CB) were subjected to CD56 magnetic-activated cell sorting (MACS) using a human CD56 MicroBead Kit, according to manufacturer instructions. (**a**) Recovery of total nucleated cells (TNC) after CD56 MACS. TNC number was determined using the trypan blue exclusion method. (**b**) Representative flow cytometry image before and after CD56 MACS. The image displays NK cells (Q1, CD3^−^CD56^+^), NKT cells (Q2, CD3^+^CD56^+^), T cells (Q3, CD3^+^CD56^−^), and other cell types (Q4, CD3^−^CD56^−^). (**c**) CD56^+^ cell percentage before and after CD56 MACS. (**d**) Efficiency of CD56 MACS. (**e**) Percentages of NK, NKT, T and other cell types before and after CD56 MACS. Results were obtained by flow cytometry and are expressed as the arithmetic mean ± SEM of 26 independent donors. A Wilcoxon test was performed, and **** indicates a *p*-value < 0.0001; (**f**) Representative flow cytometry image of the apoptosis assay. After CD56 MACS, cells were stained with FITC-Annexin V and PI. The image shows viable cells (Q4, Annexin V^−^PI^−^), early apoptotic cells (Q3, Annexin V^+^PI^−^), late apoptotic cells (Q2, Annexin V^+^PI^+^), and necrotic cells (Q1, Annexin V^−^PI^+^). (**g**) Percentages of live, early apoptotic, late apoptotic and necrotic cells before and after CD56 MACS. Results represent 19 independent donors, and are expressed as the arithmetic mean ± SEM. A Wilcoxon test was performed, and *** indicates a *p*-value of < 0.001.

An average total nucleated cell (TNC) count of (12.6 ± 1.8) × 10^6^ cells was obtained after isolation (range 1–31 × 10^6^ cells). Considering an initial MNC(CB) count of (256 ± 18) × 10^6^ cells (range 130–440 × 10^6^ cells), the overall recovery was 5.5 ± 0.6% (Fig. 1a). Following isolation, a significant enrichment of CD56^+^ NK and NKT cells was observed, with their percentage increasing from 19.3 ± 1.7% to 91.6 ± 1.5% post-MACS (Fig. 1b,c).

MACS efficiency, calculated from the proportion of CD56^+^ cells before and after enrichment, was 25.8 ± 3.3% (Fig. 1d). Representative flow cytometry images confirmed the marked expansion of the NK cell population and the concomitant depletion of non-NK subsets (Fig. 1b). Quantitative analysis further corroborates these findings, showing an isolation efficiency of 27.9 ± 3.5% for CD3⁻CD56⁺ NK cells, while only a modest enrichment of 5.6 ± 1.1% was observed for CD3^+^CD56^+^ NKT cells. Significant reductions were observed in T cells and other non-NK cell populations, which decreased from 43.8 ± 2.6% and 36.9 ± 2.5% to 1.9 ± 0.6% and 4.3 ± 0.8%, respectively (Fig. 1e). Viability assessment revealed that the majority of enriched cells remained viable (56.1 ± 4.90%, Annexin V^−^PI^−^), while 10.3 ± 1.69% were in early apoptosis (Annexin V^+^PI^−^) and 33.5 ± 5.3% in late apoptosis/necrosis (Annexin V^+^PI^+^ and Annexin V^−^PI^+^) (Fig. 1f,g).

### CD56⁺-enriched NK(CB) cells display limited effector function prior to expansion

**Fig. 2:**
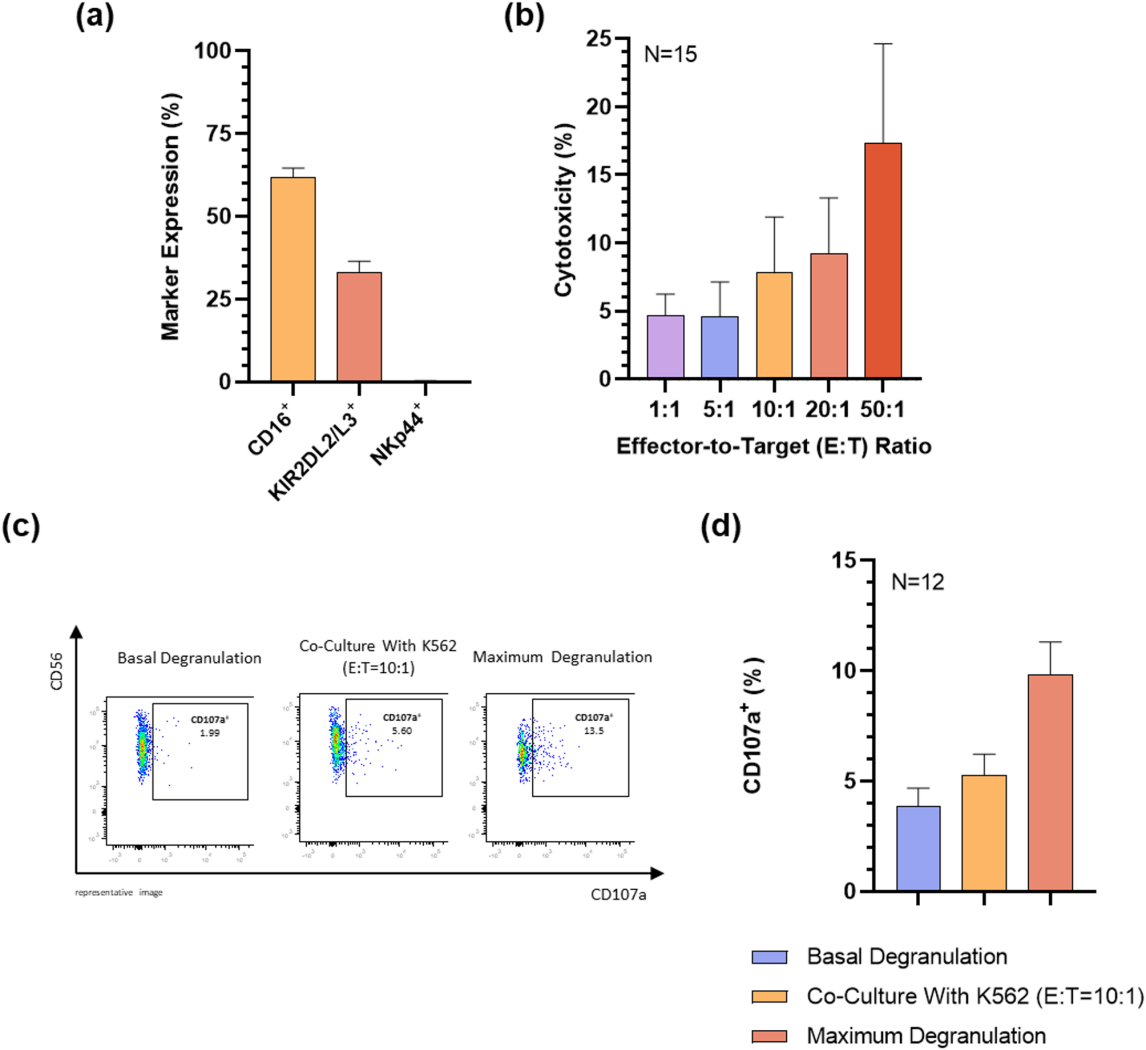
Phenotypic and functional characterization of CD56^+^-enriched cells. To assess the properties of CD56^+^-enriched cells, flow cytometry, europium release cytotoxicity assay, and degranulation assay were employed. (**a**) Expression levels of CD16, KIR2DL2/L3 and NKp44 on CD56-enriched cells assessed by flow cytometry. Results are expressed as the arithmetic mean ± SEM of 26, 18, and 18 independent donors, respectively; (**b**) Cytotoxic activity of CD56^+^-enriched cells tested using an europium release assay against K562 target cells across various E:T ratios (1:1, 5:1, 10:1, 20:1, 50:1). Results represent the arithmetic mean ± SEM, and were obtained from 15 independent donors; (**c**) Representative flow cytometry image of the CD107a degranulation assay. The degranulation capacity of CD56^+^-enriched cells was evaluated by measuring CD107a surface expression following stimulation with K562 cells at an effector-to-target (E:T) ratio of 10:1. Basal and maximum degranulation controls were included. (**d**) Expression of CD107a in CD56^+^-enriched cells, assessed by CD107a degranulation assay. Results represent the mean ± SEM from 12 independent donors.

CD16, a key marker distinguishing NK cell subsets, was expressed in 61.8 ± 2.9% of cells. Inhibitory receptors KIR2DL2/L3 were expressed on a smaller fraction, 33.0 ± 1.7%, while the activating receptor NKp44 was barely detectable, 0.3 ± 0.1% (Fig. 2a). Immediately post-isolation, CD56^+^-enriched cells exhibited low cytotoxic activity, with a maximum specific lysis of 17.3 ± 7.3% at an E:T ratio of 50:1 (Fig. 2b). Although cytotoxicity tended to increase with higher E:T ratios, the differences between tested ratios were not statistically significant. Upon stimulation with K562 cells at an E:T ratio of 10:1, only 5.8 ± 0.9% of cells expressed CD107a. Basal degranulation, measured in the absence of stimulation, was 3.8 ± 0.8%, while maximum degranulation, measured using a positive control, reached 9.3 ± 1.6% (Fig. 2c,d).

### Efficient expansion of NK cells under optimized conditions maintains phenotype and viability

**Fig. 3:**
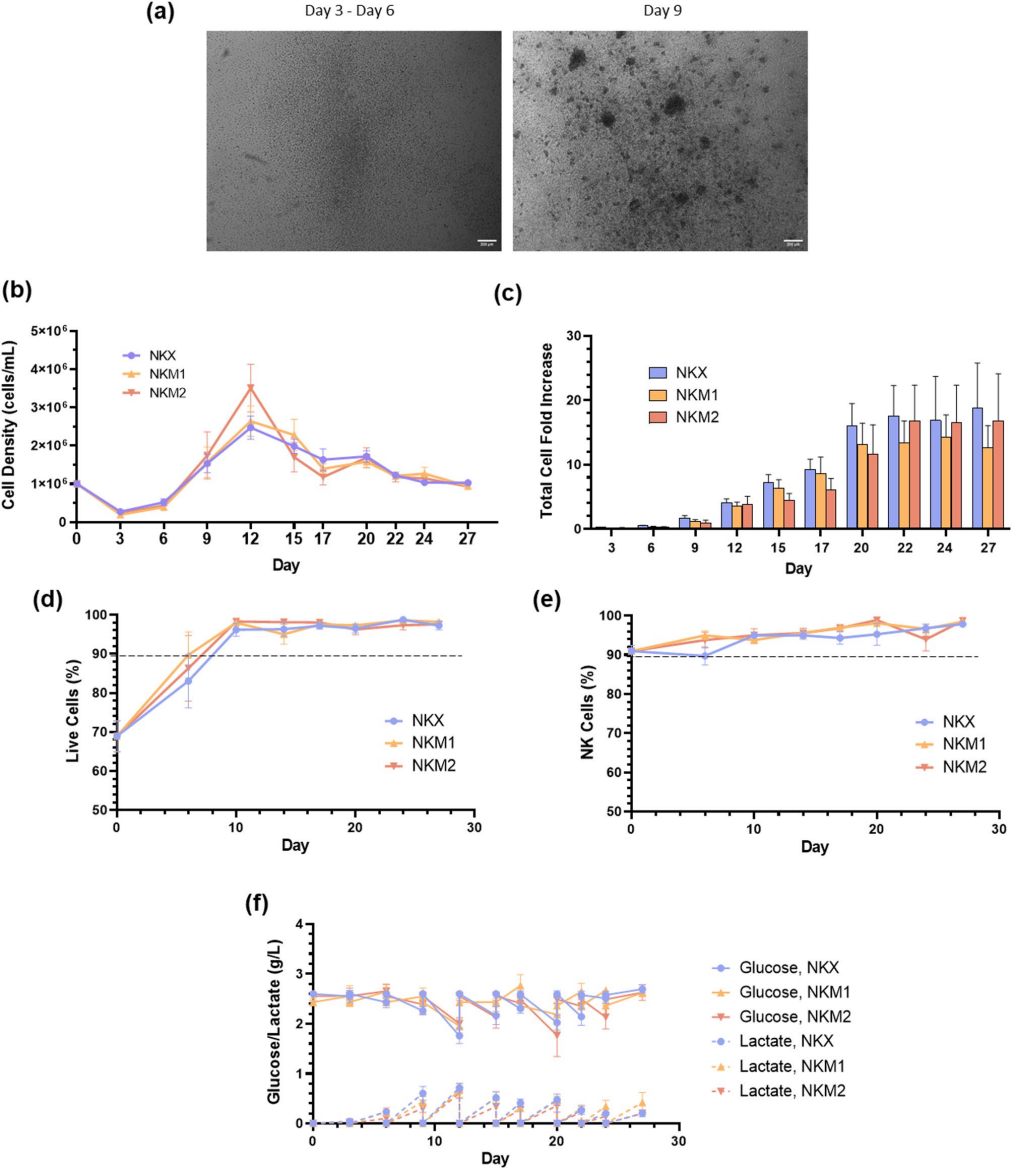
NK(CB) cell expansion using different media. After CD56 magnetic-activated cell sorting, cells were cultured using several media for comparison. The expansion results using CTS™ NK-Xpander™ (NKX) and NK MACS^®^ media (NKM1 and NKM2) are represented. (**a**) Representative bright field microscopy images showing NK(CB) cell suspension culture at the beginning of culture (days 3–6) and when proliferation begins (around day 9). (**b**) Cell density (cells/mL) throughout the culture period. (**c**) Fold increase of total cells during expansion. (**d**) Percentage of live cells and of (**e**) CD3^-^CD56 ^+^ NK(CB) cells over time, as assessed by flow cytometry. Results are represented as the arithmetic mean ± SEM. The data sets correspond to 15, 12, and 6 independent donors for NKX, NKM1, and NKM2, respectively; (**f**) Glucose and lactate concentration measurements throughout the culture period. Results represent 7, 4 and 8 independent donors, for NKX, NKM1 and NKM2, respectively. Results are expressed as the arithmetic mean ± SEM. Media compositions: NKM1: 1% Supplement + 5% human serum + 500 U/mL IL-2; NKM2: 2% Supplement + 5% human serum + 500 U/mL IL-2; NKX: 2% Supplement + 5% human serum + 500 U/mL IL-2.

Among the tested culture media, only CTS™ NK-Xpander™ (NKX) and the two NK MACS^®^ (NKM) conditions, namely NKM1 (with 1% supplement) and NKM2 (with 2% supplement), supported successful NK(CB) expansion. In contrast, GMP SCGM and X-VIVO™ 15 failed to sustain NK(CB) proliferation, while PRIME-XV NK Cell CDM and StemSpan™ SFEM II resulted in only minimal expansion, reaching approximately a 2-fold increase under the tested conditions (Fig. S2). The expansion process exhibited three distinct phases: lag phase, expansion phase and stationary phase. During the lag phase, which occurred from day 0 to day 9, no significant increase in cell numbers was observed, with a reduction in the number of plated cells on days 0–3. Representative bright-field microscopy images captured between days 3 and 6 illustrate the initial suspension culture (Fig. 3a). This was followed by the exponential phase, characterized by rapid and sustained proliferation under optimal culture conditions, including IL-2 stimulation. During this phase, NK(CB) cells tended to form multicellular clusters. Finally, the stationary phase, occurring between days 20 and 27, depending on the culture medium, was characterized by a pronounced slowdown in cell proliferation. Cell density was consistently adjusted to 1 × 10^6^ cells/mL, and it was observed a steady increase in cell numbers between days 6 and 12, corresponding to the early exponential growth phase (Fig. 3b). Among the tested culture media, NKX achieved the highest cell fold expansion, reaching 18.8 ± 7.0 by day 27, while NKM2 and NKM1 exhibited slightly lower performance, attaining maximum fold increases of 16.8 ± 7.3 and 14.3 ± 3.5, respectively, at day 27 and day 24 (Fig. 3c). However, no statistically significant differences were observed among the three conditions, indicating comparable expansion efficiencies. A notable donor-dependent variability was observed, with fold increases ranging from 3 to 98 in NKX, from 4 to 37 in NKM1 and from 2 to 45 in NKM2 (Figure S3a). When normalized to the TNC count per cord blood unit, the average fold increases corresponded to final cell yields of (237 ± 13) × 10^6^, (180 ± 6) × 10^6^, and (212 ± 13) × 10^6^ cells for the NKX, NKM1, and NKM2, respectively. Growth kinetics analysis revealed subtle differences in proliferation among the three culture media conditions. NKX exhibited a growth rate of 0.26 ± 0.01 day^−1^, corresponding to a doubling time of 2.73 ± 0.14 days. NKM1 displayed a slightly higher growth rate of 0.31 ± 0.03 day^−1^, with a doubling time of (2.39 ± 0.21) days, while NKM2 had a growth rate of 0.29 ± 0.03 day^−1^ and a doubling time of 2.52 ± 0.30 days. These results highlight only slight variations in cellular expansion kinetics, with NKM1 supporting the fastest growth, followed by NKM2 and NKX.

Throughout the culture period, all three conditions maintained high cell viability, consistently exceeding 90% from day 10 onward (Fig. 3d). The percentage of CD3^−^CD56^+^ NK cells remained high and stable during the expansion process, with a slight increase observed during the first 10 days, after which the percentages plateaued. By the end of the culture period, NK cell purity was comparable across all conditions, reaching approximately 98% (Fig. 3e). Other cell populations, such as NKT and T cells, remained negligible throughout the expansion period (less than 5%). Metabolic activity peaked between days 10 and 12, coinciding with the beginning of the exponential growth phase (Fig. 3f), and reflected by increased glucose consumption and lactate production. Importantly, lactate levels remained below the critical threshold of 20 mM^27^.

### Cytotoxicity and degranulation are enhanced in expanded NK(CB) cells

**Fig. 4:**
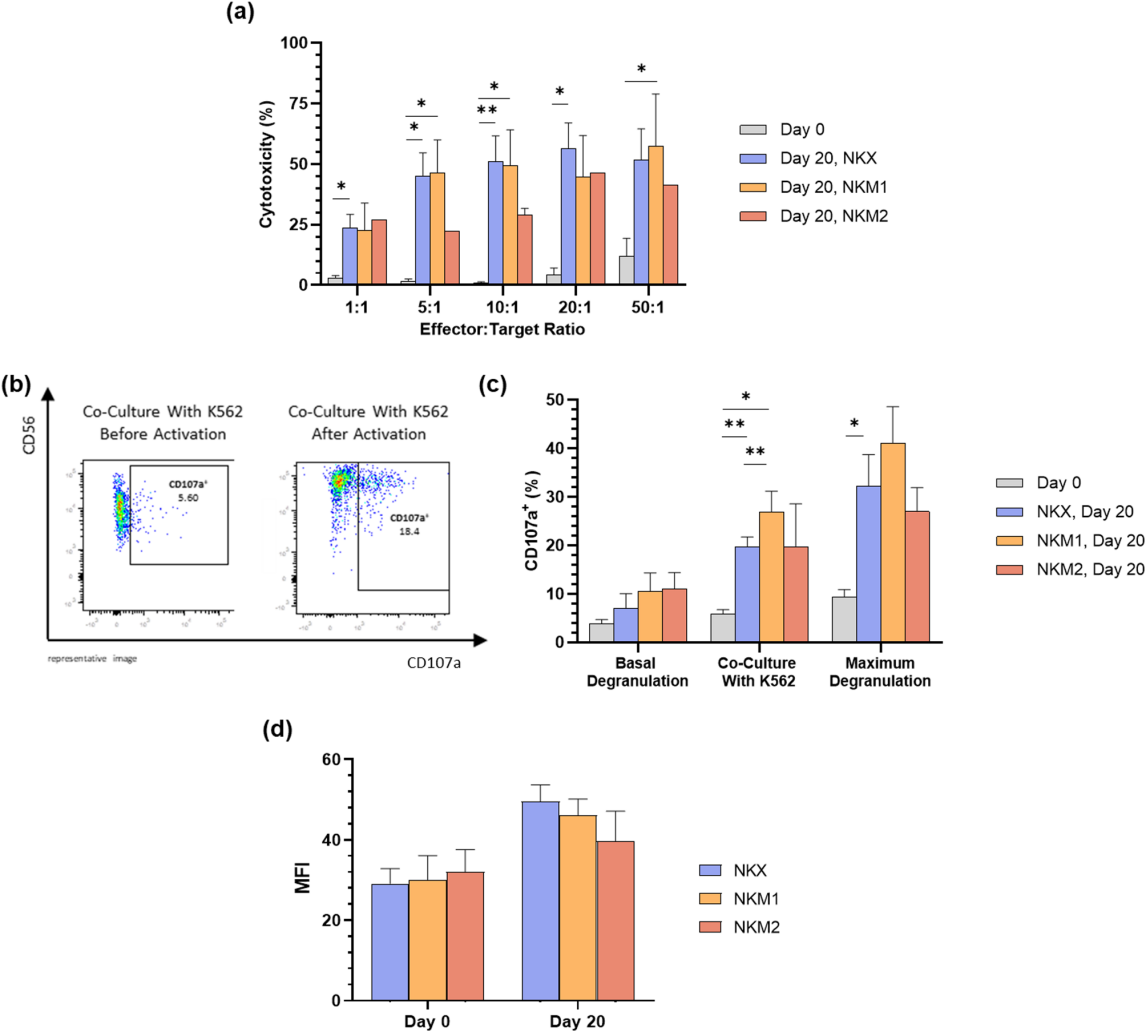
Expanded NK(CB) cell functionality. To assess the functionality of expanded NK(CB) cells, an europium release cytotoxicity assay, and degranulation assay were performed at day 20 of expansion. (**a**) Cytotoxic activity of expanded NK(CB) cells assessed using an europium release assay against K562 target cells at different effector-to-target (E:T) ratios (1:1, 5:1, 10:1, 20:1, 50:1). Results represent the arithmetic mean ± SEM from 13, 7 and 2 independent donors for NKX, NKM1 and NKM2, respectively. Paired t-tests were performed, * and ** indicate *p*-value of < 0.05 and < 0.01, respectively; (**b**) Representative flow cytometry plots of the CD107a degranulation assay before and after activation and expansion. The degranulation capacity of expanded NK(CB) cells was evaluated by measuring CD107a surface expression following stimulation with K562 cells at an E:T ratio of 10:1. (**c**) Expression of CD107a in expanded NK(CB) cells, assessed by CD107a degranulation assay. (**d**) Mean fluorescence intensity (MFI) of CD107a in CD107a^+^ NK(CB) cells at day 20 of expansion. Results represent the arithmetic mean ± SEM from 9, 6 and 3 independent donors for NKX, NKM1 and NKM2, respectively. Wilcoxon and paired t-tests were performed, and * and ** indicate a *p*-value of < 0.05 and < 0.01, respectively.

A significant increase in NK cell-mediated lysis of target cells was observed post-expansion across all tested culture media and at each E:T ratio. Despite this enhancement, no statistically significant differences in cytotoxicity levels were observed among the different culture conditions. A notable increase in target cell lysis occurred between the 1:1 and 5:1 E:T ratios, after which the percentage of lysis plateaued at higher ratios. At an E:T ratio of 5:1, the cytotoxicity reached 45.1 ± 9.5% for NKX, 45.1 ± 13.7% for NKM1, and 24.2 ± 1.8% for NKM2 (Fig. 4a). Regarding degranulation potential, representative flow cytometry analysis showed an increased CD107a expression in expanded cells compared to freshly isolated CD56^+^-enriched cells (Fig. 4b). Quantitative analysis confirmed an overall increase in the percentage of CD107a^+^ cells post-expansion, with values of 19.7 ± 2.0% for NKX, 26.9 ± 3.5% for NKM1, and 19.8 ± 8.8% for NKM2, without statistically significant differences across all tested conditions (Fig. 4c). Additionally, the mean fluorescence intensity (MFI) of CD107a in CD107a^+^ NK(CB) cells increased relative to day 0 by about 1.7-fold for NKX, 1.5-fold for NKM1, and 1.3-fold for NKM2 (Fig. 4d).

### Activation-driven phenotypic changes were observed in expanded NK(CB) cells

**Fig. 5:**
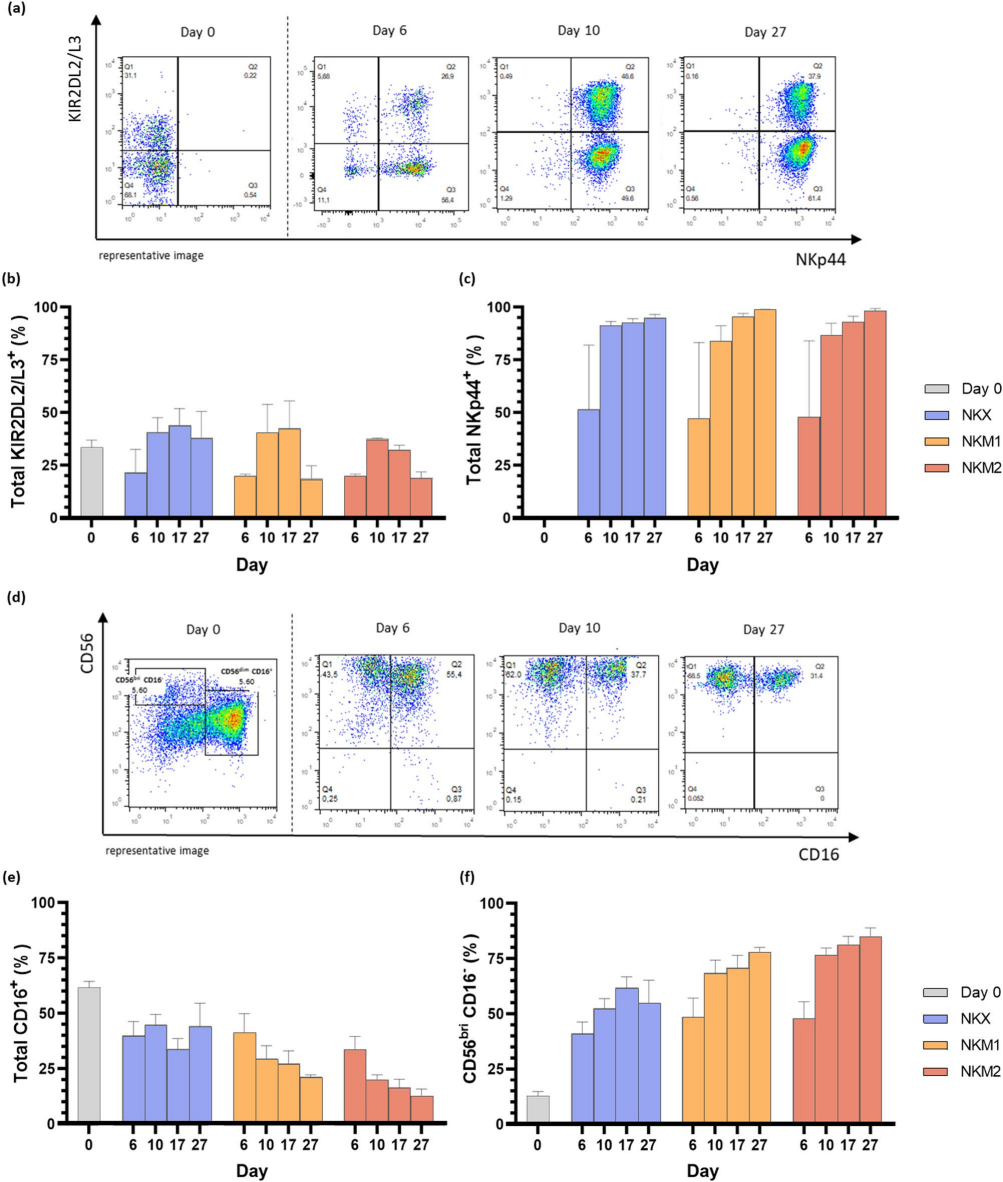
Characterization of NK(CB) cells over a 27-day culture period. This figure summarizes the flow cytometry phenotypic characterization of NK(CB) cells during a 27-day culture period across different media conditions (NKX, NKM1, and NKM2). (**a**) Representative flow cytometry plots illustrating the expression of the activating receptor NKp44 and the inhibitory receptors KIR2DL2/L3 on days 0, 6, 10 and 27 of culture. (**b**) Total expression of inhibitory receptors KIR2DL2/L3 (Q1:KIR2DL2/L3^+^NKp44^−^ and Q2:KIR2DL2/L3^+^NKp44^+^) and (**c**) activation receptor NKp44 (Q2:KIR2DL2/L3^+^NKp44^+^ and Q3:KIR2DL2/L3^−^NKp44^+^) markers throughout the expansion period. Results indicate the arithmetic mean ± SEM, based on data from 11, 6 and 6 independent donors, for NKX, NKM1 and NKM2, respectively; (**d**) Representative flow cytometry plots showing the expression of CD56 and CD16 expression on days 0, 6, 10 and 27. (**e**) Total expression of CD16 (Q2:CD56^+^CD16^+^ and Q3:CD56^−^CD16^+^) and of (**f**) CD56^bri^CD16^+^ NK cell subpopulations at different time points during expansion. Results are expressed as the arithmetic mean ± SEM, from 15, 12 and 6 independent donors, for NKX, NKM1 and NKM2, respectively.

The expression of NKp44, an activating receptor, and KIR2DL2/L3, inhibitory receptors, was monitored throughout time to evaluate activation dynamics (Fig. 5a). Total KIR2DL2/L3 expression remained stable at approximately 30% (Fig. 5b), while total NKp44 expression increased significantly in activated and expanded cells, reaching more than 90% from day 10 onwards (Fig. 5c). The expression of CD16, a key marker for NK cell antibody-dependent cell-mediated cytotoxicity (ADCC), was also evaluated, revealing, two distinct NK cell subpopulations: the CD56^bright(bri)^CD16^−^ subset and the CD56^dim^CD16^+^ subset (Fig. 5d). Interestingly, CD16 expression remained stable from day 6 onwards under NKX culture conditions, but progressively declined in NKM1 and NKM2 culture media (Fig. 5e). This decline was accompanied by a significant shift in population dynamics, marked by the disappearance of the CD56^dim^CD16^+^ subset and an increase in the CD56^bri^CD16^−^ population (Fig. 5f). NKp44 upregulation reflects activation rather than developmental maturation, and the shift toward CD56^bri^CD16⁻ subsets in NKM1 and NKM2 is consistent with cytokine-driven phenotypes.

### NK(CB) cell expansion costs vary significantly among culture media

**Fig. 6:**
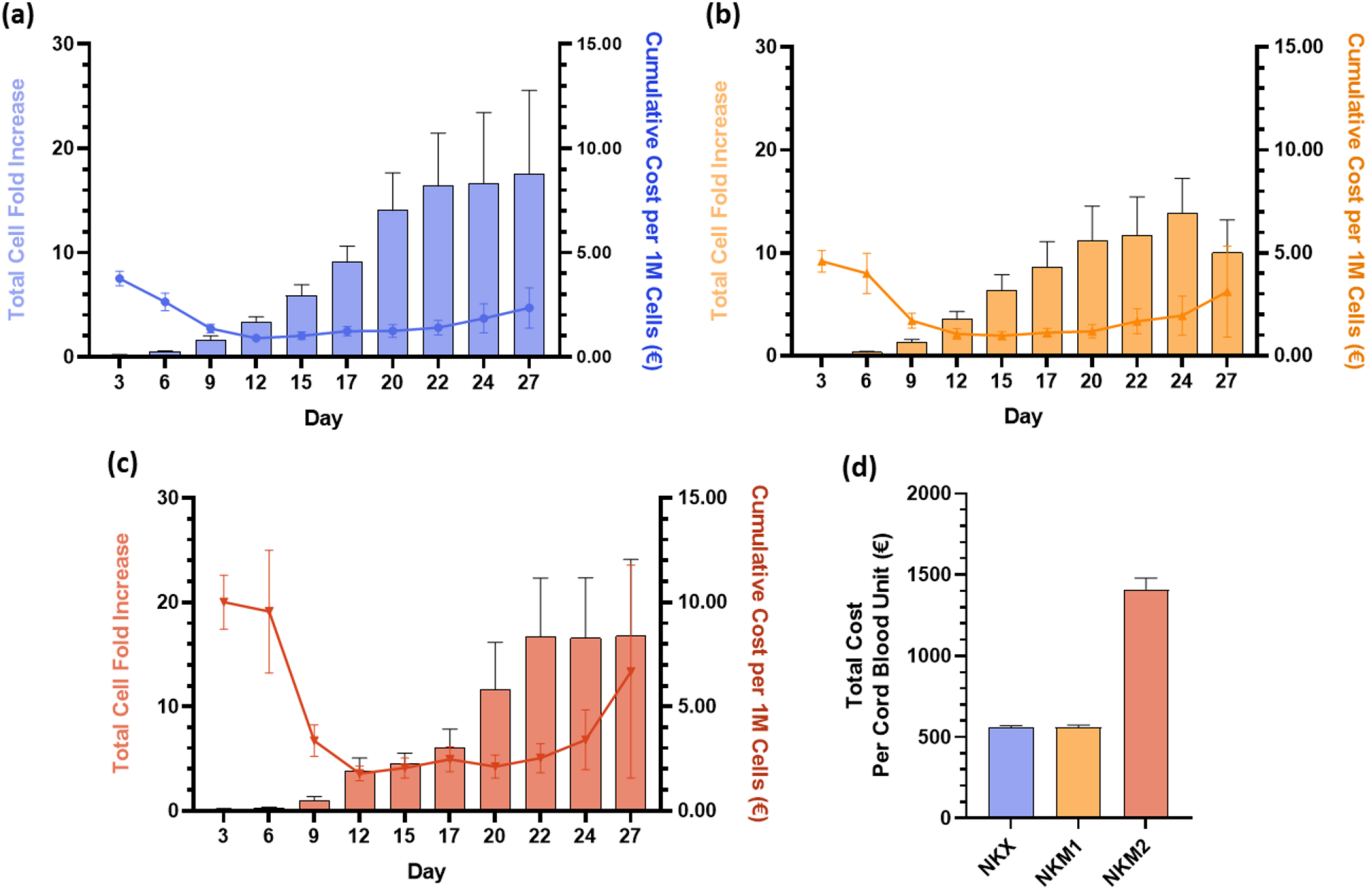
Total cumulative costs during NK(CB) cell expansion across the three evaluated media: NKX, NKM1, and NKM2. Total cell fold increase (left axis) and cumulative cost per 1 million cells produced (right axis) for (**a**) NKX, (**b**) NKM1, and (**c**) NKM2. Results are represented as the arithmetic mean ± SEM. The data sets include 15, 12, and 6 independent donors for NKX, NKM1, and NKM2, respectively. (**d**) Total cost per cord blood unit for NKX, NKM1, and NKM2, enabling direct comparison of overall manufacturing cost.

Total cumulative costs were analyzed and revealed comparable cost per million cells produced between NKX and NKM1, with values of 2.4 ± 1.0 € and 3.1 ± 2.2 €, respectively (Fig. 6a,b). In contrast, the cost associated with NKM2 medium was significantly higher, reaching 6.7 ± 5.1€ (Fig. 6c). When considering the final cell yields, the total average cost per cord blood unit was calculated as 557 ± 13 € for NKX, 560 ± 14 € for NKM1, and 1410 ± 70 € for NKM2 (Fig. 6d).

## Discussion

NK cells are increasingly used in immunotherapeutic strategies to treat hematologic malignancies and solid tumors due to their intrinsic cytotoxicity without prior sensitization. NK cells from CB are particularly attractive for allogeneic adoptive cell therapy because their immature receptor profile^5^ reduces the risk of GvHD, while maintaining proliferative and anti-tumor functions^3,28^. Moreover, CB is an ethically accepted, readily available source already established for hematopoietic stem cell transplantation^7^, making it ideal for off-the-shelf cell-based therapies^3,4^. However, the limited NK cell yield per CB unit remains a major challenge for direct clinical application^3^. NK(CB) cells account for up to 30% of MNCs^29^, corresponding to an average of 70–80 million NK cells per CB unit^30^. Even with a theoretical 100% isolation efficiency, this number remains far below the doses required for allogeneic transplantation^31^. This limitation underscores the need for efficient ex vivo expansion strategies, that not only increase cell yields, but also enhance cytotoxic functionality^2^. In this study, we evaluate different expansion media for NK(CB) cells under static feeder-free conditions, assessing their impact on immunophenotype, cytotoxic function and cost-effectiveness.

CD56^+^ cell enrichment was performed using MACS, a rapid and flexible approach^32^. This strategy provides a practical alternative to extended CD3^+^ depletion protocols combined with feeder cells, which typically require up to 21 days^33,34^. Our protocol achieved a 5-fold increase in CD56^+^ purity but resulted in < 30% NK recovery with high donor variability. These values are within the wide range reported for NK(PB) recovery, which varies from 13%^35^ to 90%^36^. Recovery efficiency depends strongly on the initial NK cell frequency and bead saturation, which can be optimized by adjusting bead-to-cell ratios, performing sequential selection rounds, or employing alternative strategies such as FACS sorting^37,38^. For example, a 36% NK(PB) recovery rate using combined CD3^−^/CD56^+^ selection is comparable to our findings^39^. A known limitation of MACS is its potential to induce cell death via mechanical stress^32^, reflected by the reduced viability post-isolation. However, the subsequent culture improved cell viability, indicating that NK(CB) cells adapted to ex vivo conditions.

Following CD56^+^ enrichment, efficient expansion becomes crucial to overcome the low recovery. However, few protocols are specifically optimized for NK(CB) expansion; most are adapted from NK(PB) methodologies relying on feeder cells^28,40^ or intensive cytokine stimulation^41^. In this study, we compared two feeder-free culture media, NKM and NKX, selected from an initial screen of six candidates, evaluating each under conditions consistent with their commonly reported or manufacturer-recommended usage. Their ability to support NK(CB) proliferation, functional activation, and cost-efficiency were evaluated. NKM was tested in two formulations with different supplement concentrations, based on previous studies^17,22^. Although many expansion protocols combine multiple cytokines to enhance NK growth and activation^2,3^, IL-2 alone remains well-documented as sufficient to promote NK proliferation and cytotoxic potential^28^. Moreover, feeder-free systems offer significant translational advantages by minimizing contamination risks, reducing variability and simplifying downstream processing, facilitating compliance with GMP guidelines^42^. NK(CB) cells cultured in NKM and NKX followed a typical growth curve. The initial lag phase was characterized by lower cell viability and reduced cell numbers, reflecting adaptation to ex vivo conditions. During this period, cell aggregation was observed, a phenomenon previously described as enhancing IL-2 receptor signaling and promoting NK cell activation and proliferation^43^. This highlights an intrinsic limitation of feeder-free systems, where NK cells depend solely on cytokines in the culture medium and lack natural cell-to-cell interactions that physiologically support expansion^44^. Nevertheless, the gradual improvement in cell viability during the lag phase enabled a successful exponential growth phase (days 9–20), with marked increases in total cell numbers (to around 200 × 10^6^ TNC per cord blood), high viability and sustained CD56^+^ purity. While the maximum fold expansion was comparable between media, NKX demonstrated slightly higher consistency and predictability across donors. Glucose consumption and lactate production paralleled cell proliferation dynamics and no nutrient limitations were detected, confirming that the applied feeding regimen was adequate^27^. Still, the overall fold expansion remained lower than typically achieved with feeder-based systems^33,34^. To address this limitation, a factorial design experiment could help identify cost-effective cytokine combinations that maximize proliferation^45^. Future studies should also normalize cytokine and serum conditions across culture media, as such standardization would allow a more precise assessment of the intrinsic properties of each basal culture medium.

Immediately post-isolation, NK(CB) cells displayed low cytotoxic activity and modest CD107a degranulation, together with low NKp44 and intermediate KIR2DL2/L3 expression. These findings indicate limited effector function at baseline. By day 20, NK(CB) cells showed clear functional activation across all conditions, including increased NKp44 expression and enhanced degranulation, consistent with the acquisition of a more cytotoxic profile^46^. Given the restricted phenotypic panel used in this study, these activation-driven changes and subset redistributions do not allow conclusions regarding developmental maturation. Notably, NKX cultures displayed a higher proportion of CD16⁺ cells, which is particularly relevant for ADCC^47^. For a more comprehensive assessment of maturation, activation and functional competence, future studies should include additional receptors such as NKp46, NKG2D, DNAM-1 and TRAIL (activating), as well as NKG2A and KLRG1 (inhibitory), among others^48^, as the restricted phenotypic panel used here represents a limitation of the study. These results suggest that NKM may preferentially support the expansion or survival of regulatory CD56^bri^CD16⁻ cells. The concurrent decline in CD16 and KIR2DL2/L3 expression during the stationary phase in NKM cultures implies that culture conditions can influence NK cell subset composition over time, with potential consequences for functional potency. Mechanistically, the reduction in CD16 is consistent with cytokine-driven downregulation previously described in high-IL-2 environments^49^, reflecting a shift toward a more CD56^bri^ phenotype. From a therapeutic perspective, this trend may reduce ADCC capacity, underscoring the need to optimize cytokine exposure and define an appropriate harvest window to balance expansion, activation and effector function. The significant increase in CD107a^+^ cells upon stimulation further correlated with enhanced cytotoxic potential. Taken together, these longitudinal trends are more consistent with cytokine-driven activation and subset selection than with progressive differentiation or functional exhaustion.

Cost analysis revealed that NKM1 and NKX had comparable costs, higher during the lag phase but stabilizing during exponential growth. In contrast, the stationary phase contributed disproportionately to overall costs without significant gains in cell yield, emphasizing the need to define an optimal culture endpoint, potentially around day 24. NKM2, requiring double supplementation, was approximately twice as expensive as NKM1, reinforcing the importance of balancing cell yield with cost-efficiency when selecting feeder-free media for NK(CB) expansion. This comparison also showed that, despite similar cost per million cells, the overall manufacturing cost per cord blood unit was substantially higher for NKM2 than for NKX or NKM1, due to supplement concentration and cumulative medium consumption.

Donor variability had a substantial impact on expansion success, particularly during the lag phase, where early cell death often determined the overall outcome. Donors that performed well in NKX tended to also expand efficiently in NKM, and the same trend was observed for poor performers, suggesting donor-intrinsic determinants. Exploratory analyses did not reveal meaningful correlations between baseline NK(CB) potency or initial TNC/NK content and subsequent expansion or cytotoxicity outcomes, underscoring the strong intrinsic inter-donor variability. To improve the predictability of expansion outcomes in a manufacturing setting, integrating Process Analytical Technology (PAT) tools may prove valuable. Real-time monitoring of cell adaptation and metabolic activity could allow early detection of suboptimal trajectories and enable timely process adjustments^47,50^. Such tools could also help identify critical process attributes, including NK cell frequency, maturation state, or cytokine responsiveness, that may support donor-selection strategies and enhance manufacturing consistency and clinical efficacy^47,50^.

Taken together, our findings indicate that while all three conditions supported NK(CB) proliferation under feeder-free conditions, NKX demonstrated greater donor-to-donor consistency, preserved cytotoxic CD16^+^ subsets and sustained functional activation, features that may be advantageous for applications requiring strong ADCC responses. In contrast, NKM, particularly in the standard supplementation condition, favored CD56^bri^CD16⁻ regulatory-like profiles and may therefore be more suitable for immunomodulatory contexts. Although both culture media enabled robust expansion, NKX showed a more predictable performance across donors, which could facilitate translational implementation where manufacturing reproducibility is critical.

## Conclusion

This study demonstrates that feeder-free, IL-2-supplemented culture systems can efficiently support the ex vivo expansion of cord blood–derived NK cells, maintaining high viability and functional cytotoxicity. Among the evaluated culture media, NK-Xpander™ (NKX) consistently achieved superior and more predictable expansion across donors, while the NK MACS^®^-based formulations (NKM1 and NKM2) supported robust proliferation with distinct phenotypic outcomes. NKX preserved cytotoxic CD16^+^ subsets, making it especially suitable for applications requiring strong ADCC, whereas NKM formulations tended to favor CD56^bri^CD16^−^ regulatory-like profiles, which may be advantageous in immunomodulatory contexts. The use of cord blood as a cell source offers additional benefits, including ethical accessibility, lower immunogenicity and high proliferative potential, supporting its use in allogeneic and off-the-shelf immunotherapies. Altogether, these findings provide a foundation for standardized, scalable and economically sustainable manufacturing strategies, advancing the development of NK(CB)- and CAR-NK cell-based therapies.

## Supplementary Information

Below is the link to the electronic supplementary material.

Supplementary Material 1

## Data Availability

The data that support the findings of this study are available on request from the corresponding author.
