# Supplementary material for: Feeder-free ex vivo expansion of cord blood-derived natural killer cells for enhanced proliferation and functional maturation
Source: Sci Rep. 2026 Feb 24;16:10417. doi: 10.1038/s41598-026-41101-5 (PMC13031528; doi:10.1038/s41598-026-41101-5)
Supplement: Supplementary file 1 — Supplementary Material 1 [file 41598_2026_41101_MOESM1_ESM.docx]

**Supplementary information**

Table S1 - Complete NK cell culture media compositions. All culture media were sterilized by filtration using a 0.22 μm filter. A/A: Antibiotic-Antimycotic; IL-2: Interleukin-2.

| **Name** | **Composition** | **Reference** |
| --- | --- | --- |
| **CTS^™^ NK-Xpander^™^** | 92% (v/v) Basal Medium | A5019001, Thermo Fisher Scientific |
|  | 2% (v/v) Medium Supplement | A5019001, Thermo Fisher Scientific |
|  | 5% (v/v) Human Serum | H6914, Sigma-Aldrich or S4190, Biowest |
|  | 1% (v/v) A/A | 15240062, Thermo Fisher Scientific |
|  | 500 U/mL IL-2 | 200-02, Peprotech |
| **GMP SCGM** | 99% (v/v) Basal Medium | 20802-0500, Sartorius CellGenix GmbH |
|  | 1% (v/v) A/A | 15240062, Thermo Fisher Scientific |
|  | 1000 U/mL IL-2 | 200-02, Peprotech |
| **NK MACS^®^** | 93% (v/v) or 92% (v/v) Basal Medium | 130-114-429, Milteny Biotec |
|  | 1% (v/v) or 2% (v/v) Medium Supplement | 130-114-429, Milteny Biotec |
|  | 5% (v/v) Human Serum | H6914, Sigma-Aldrich or S4190, Biowest |
|  | 1% (v/v) A/A | 15240062, Thermo Fisher Scientific |
|  | 500 U/mL IL-2 | 200-02, Peprotech |
| **PRIME-XV NK Cell CDM** | 99% (v/v) Basal Medium | 91215, FUJIFILM Irvine Scientific |
|  | 1% (v/v) A/A | 15240062, Thermo Fisher Scientific |
|  | 1000 U/mL IL-2 | 200-02, Peprotech |
| **StemSpan^™^ SFEM II** | 99% (v/v) Basal Medium | 09605, STEMCELL Technologies |
|  | 1% (v/v) A/A | 15240062, Thermo Fisher Scientific |
|  | 1000 U/mL IL-2 | 200-02, Peprotech |
| **X-VIVO^™^ 15** | 99% (v/v) Basal Medium | 02-060Q, Lonza |
|  | 1% (v/v) A/A | 15240062, Thermo Fisher Scientific |
|  | 1000 U/mL IL-2 | 200-02, Peprotech |

Table S2 - Cost analysis of reagents used in “NKX” and “NKM” complete culture media. The “Total Cost” accounts for the dilution factors and proportional use of each component in the preparation of the respective complete culture media.

| **Condition** | **Reagent** | **Unit Price**  **(€)** | **Quantity** | **Total Cost (€/mL)** |
| --- | --- | --- | --- | --- |
| NKX | CTS^™^ NK-Xpander^™^ Medium (including supplement) | 203,00 | 1 | 0,45 |
|  | Human Serum AB Male HIV tested | 424,00 | 1 |  |
|  | Antibiotic-Antimycotic (100X) | 73,50 | 1 |  |
|  | Human IL-2 Recombinant Protein | 802,00 | 1 |  |
| NKM1 | NK MACS^®^ Medium (including supplement) | 202,00 | 1 | 0,45 |
|  | Human Serum AB Male HIV tested | 424,00 | 1 |  |
|  | Antibiotic-Antimycotic (100X) | 73,50 | 1 |  |
|  | Human IL-2 Recombinant Protein | 802,00 | 1 |  |
| NKM2 | NK MACS^®^ Medium (including supplement) | 202,00 | 2 | 0,85 |
|  | Human Serum AB Male HIV tested | 424,00 | 1 |  |
|  | Antibiotic-Antimycotic (100X) | 73,50 | 1 |  |
|  | Human IL-2 Recombinant Protein | 802,00 | 1 |  |


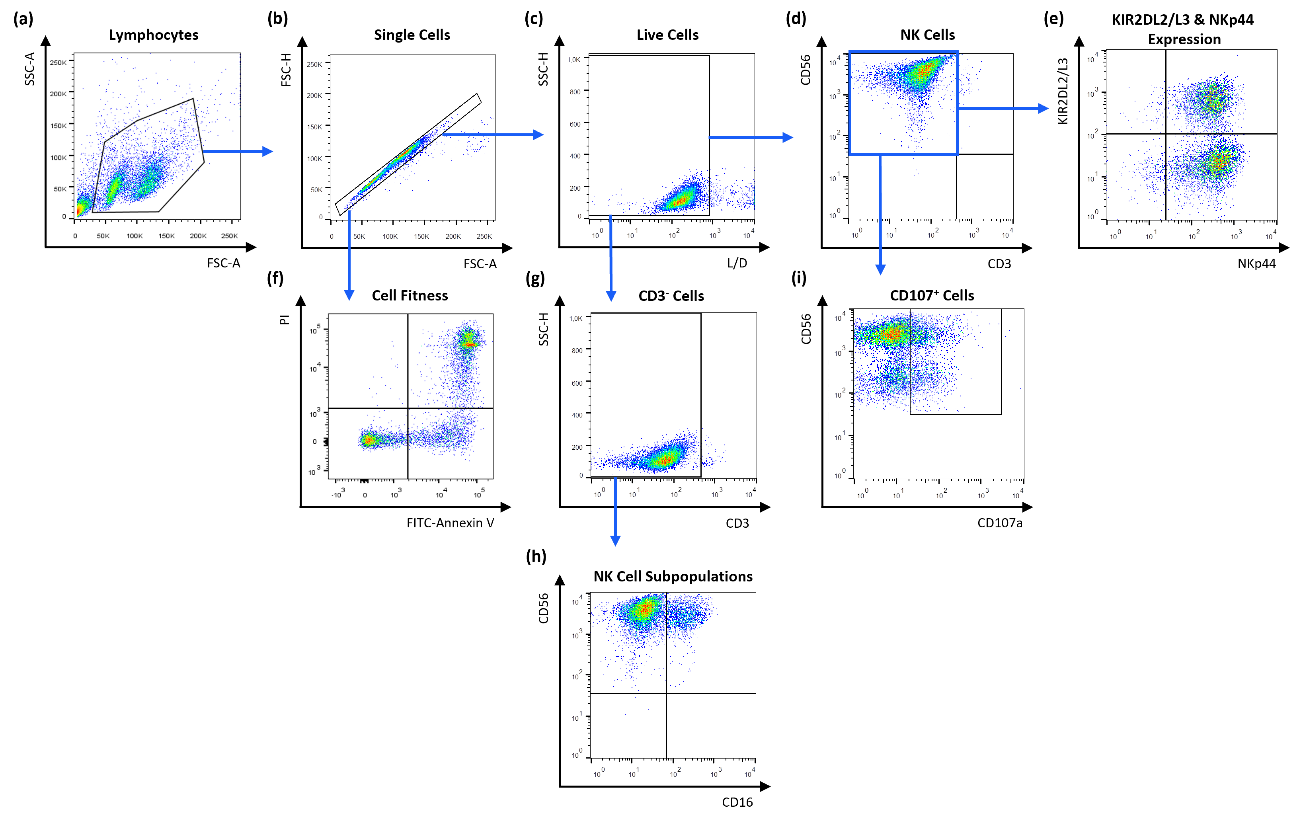
Figure S1- Flow cytometry gating strategy for umbilical cord blood (CB) natural killer (NK) cells. This figure outlines the step-by-step gating strategy used to identify and analyze NK cells derived from CB via flow cytometry. (a) Lymphocytes: The lymphocyte population was gated based on side scatter area (SSC-A) and forward scatter area (FSC-A). (b) Single Cells: Single cells were selected by comparing forward scatter area (FSC-A) with forward scatter height (FSC-H). (c) Viability Assessment: Live cells were identified using the LIVE/DEAD (L/D) viability stain, detected on the APC or APC-Cy7 channel. (d) NK Cell Identification: Surface markers CD3 and CD56, conjugated to PerCP-Cy5.5 and PE, respectively, were used to define distinct cell populations on a CD3 vs. CD56 plot (NK cells (CD3^–^CD56^+^), NKT cells (CD3^+^CD56^+^), T cells (CD3^+^CD56^-^), other cell types (CD3^–^CD56^-^)). (e) NK Cell Activation/Inhibitory Profile: The NK cell population was examined for the expression of NKp44 and KIR2DL2/L3 surface markers, labeled with APC and FITC fluorescent dyes, respectively. (f) Cell Fitness: FITC-Annexin V apoptosis assay was used to evaluate cell fitness by distinguishing different cell populations, including viable cells (FITC-Annexin V⁻PI⁻), early apoptotic cells (FITC-Annexin V⁺PI⁻), late apoptotic cells (FITC-Annexin V⁺PI⁺), and necrotic cells (FITC-Annexin V⁻PI⁺). (g) CD3^-^ Population: Gating on CD3^-^ cells allowed precise analysis of NK cell subpopulations. (h) NK Cell Subpopulations Analysis: CD16, conjugated to FITC, was used to distinguish NK subsets: CD56^bri^CD16^-^ and CD56^dim^CD16^+^. (i) Degranulation Detection: Degranulating NK cells were identified by the surface expression of CD107a, labeled with FITC.


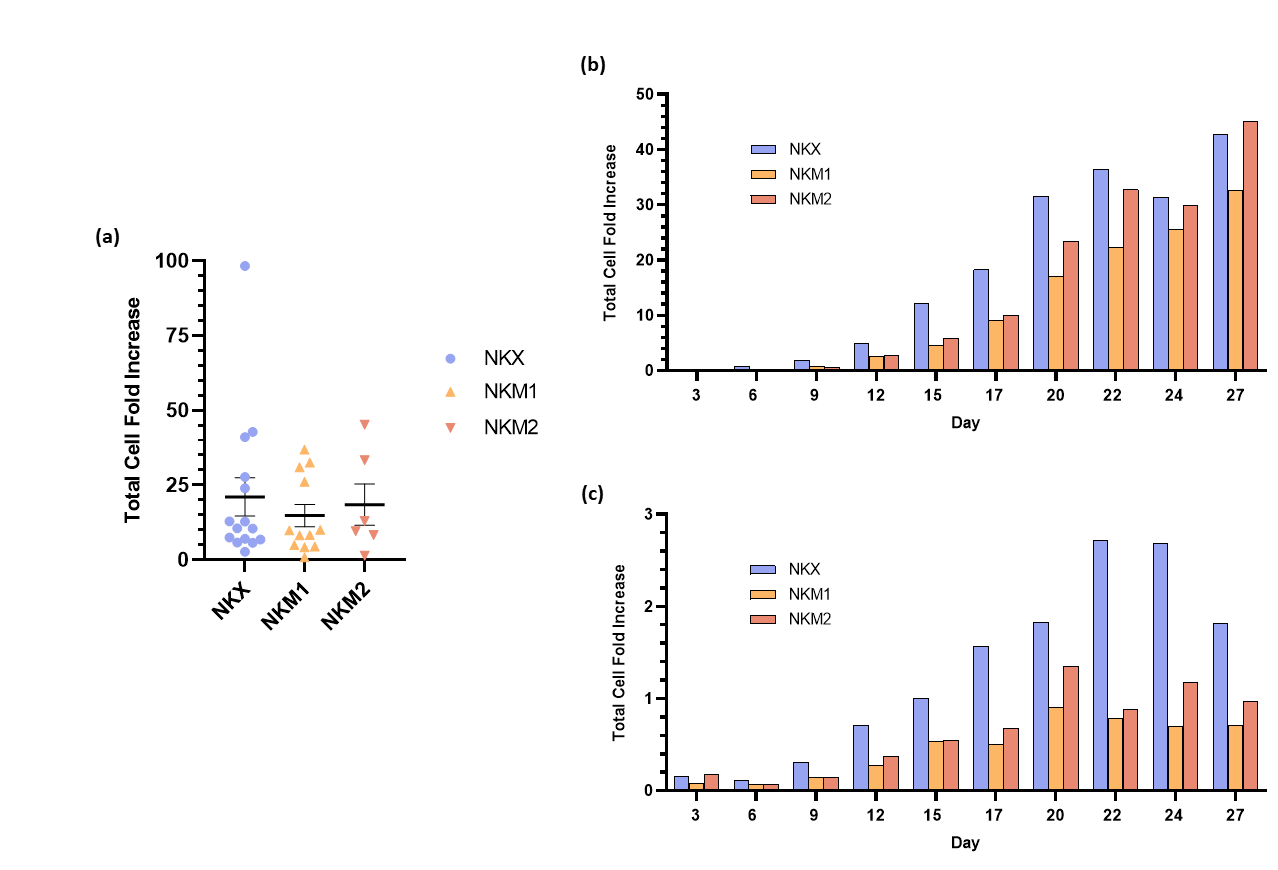
**Figure S2 - Fold increase of total cells during expansion using different media.**
Following CD56 magnetic-activated cell sorting, cells were cultured in various media to evaluate their impact on cell proliferation. This figure presents the fold increase in total cell numbers using GMP SCGM, X-VIVO^™^ 15, PRIME-XV NK Cell CDM, and StemSpan^™^ SFEM. Results are expressed as the mean ± SEM from 3 independent donors.

**Figure S3 - Fold increases achieved with each medium.** (a) Maximum fold increase obtained using CTS^™^ NK-Xpander^™^ (NKX) and NK MACS^®^ media (NKM1 and NKM2). Results represent data from 15, 12, and 6 independent donors for NKX, NKM1, and NKM2, respectively, and are expressed as mean ± SEM. (b) Donor exhibiting the highest fold increase across all three media. (c) Donor exhibiting the lowest fold increase across all three media.
